# Differences in functional block during extrastimulation from the right atrial appendage and Bachmann bundle region; a potential mechanism of observed anti-fibrillatory effects of Bachmann bundle pacing

**DOI:** 10.1093/europace/euag117

**Published:** 2026-05-11

**Authors:** Brett D Atwater, Mutaz Alkalbani, Zachary Hollis

**Affiliations:** Section of Cardiac Electrophysiology, Inova Schar Heart and Vascular, Falls Church, VA, USA; Section of Cardiac Electrophysiology, Inova Schar Heart and Vascular, Falls Church, VA, USA; Section of Cardiac Electrophysiology, Inova Schar Heart and Vascular, Falls Church, VA, USA

**Keywords:** Atrial fibrillation, Effective refractory period, Conduction velocity, Bachmann bundle pacing

## Introduction

Atrial pacing improves the quality of life in patients with sinus node dysfunction, but a higher atrial pacing burden is associated with a higher frequency of atrial fibrillation (AF) events.^1–4^ Competitive atrial pacing (CAP) may cause closely coupled premature atrial contractions (PACs) that are responsible for a significant proportion of pacemaker-detected AF episodes. Bachmann bundle (BB) pacing has been associated with a reduction in AF burden compared to right atrial appendage (RAA pacing).^5,6^ Pacing from a location with a narrow vulnerable gap between the relative refractory period (RRP) and effective refractory period (ERP) may reduce the probability that CAP occurs within the vulnerable gap, thereby reducing the probability that CAP induces AF. We hypothesized that the BB region may have a narrower vulnerable gap than the RAA, sought to identify areas of atrial conduction delay produced by PACs delivered within the vulnerable gap from the BB vs. RAA, and compared the incidence of AF inducibility with extrastimulation from the RAA and BB in a population with AF.

## Methods

### Population

In total, 19 consecutive patients with a history of AF and no prior ablation procedures scheduled for AF ablation were included if they had an uninterrupted sinus rhythm for ≥48 h prior to the procedure. Class I or III antiarrhythmic drugs were held ≥ 5 half-lives before the procedure, except amiodarone, which was held ≥ 2 weeks.

### Procedure

General anaesthesia was provided, and a quadripolar catheter was placed in the left atrial appendage, a decapolar catheter was placed in the coronary sinus, and a quadripolar catheter was sequentially positioned in random order in the BB and RAA. The BB was identified in 19/19 patients as the location in the RA/superior vena cava junction with a far field RA signal, a near field BB potential, where pacing produced a tall *P* wave with a duration 10 ms shorter than sinus rhythm in the inferior leads. Single extrastimulation was provided at twice the diastolic threshold with 10 ms increments with a drive train of 8 beats at 600 ms. The RRP was defined as the longest S1/S2 interval that produced delay to any other atrial electrogram, ERP was defined as the longest S1/S2 interval that failed to capture, and the vulnerable gap was defined as the difference between the RRP and ERP. Right atrial conduction time (RACT) was calculated as the interval from the stimulation artifact to the ostial CS electrogram. Left atrial conduction time (LACT) was calculated as the interval from the ostial CS EGM to the latest LA electrogram. To validate the RACT and LACT measurements, complete RA and LA activation maps were generated after the last drive beat and the S2 10 ms above ERP from BB and RAA in a subset of 3/19 subjects. Induced arrhythmias, including PACs, atrial tachycardia, atrial flutter, and AF, were annotated. The site of arrhythmia initiation was annotated as the location of the electrogram with the earliest activation during the initiating beat. The median (25th percentile, 75th percentile) ERP, RRP, and vulnerable gap were compared between BB and RAA pacing locations using Wilcoxon rank tests. RACT and LACT were compared after the drive train and S2, 10 ms above ERP. The protocol was approved by the IRB with a waiver of consent and HIPAA authorization (IRB # INOVA-2024-348).

## Results

**Figure 1:**
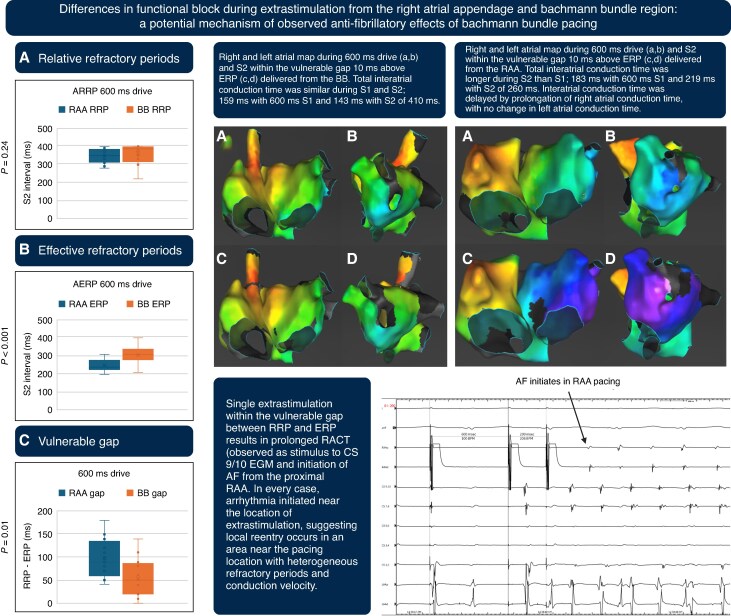
Comparisons of atrial relative refractory period (ARRP), atrial effective refractory period (AERP), and vulnerable gap between right atrial appendage (RAA) and Bachmann bundle (BB) pacing locations. Electro-anatomical maps of the right and left atrium after the last beat of the drive train of 600 ms (*A*,B) and after the S2 10 ms above ERP (*C*,D) from the BB (left) and RAA (right). Total interatrial conduction time is more prolonged after RAA than BB extrastimulation, with conduction delay occurring exclusively within the RA. Intracardiac electrograms during extrastimulation from the RAA show that AF initiates at the electrode located at the site of pacing. This was observed in 84/84 induced arrhythmias.

Nineteen patients were included (mean age 67 ± 10 years, P wave duration 145 ± 10 ms, PR interval 205 ± 72 ms, left atrial volume indexed 38 ± 14 mL/m^2^.) The RRP was similar when S1 and S2 were delivered from the RAA 350 (310,380) ms or the BB 385 (325,385) ms, *P* = 0.24. The ERP was significantly lower from the RAA 240 (200,310) ms than the BB 310 (210,400) ms, *P* < 0.001. The vulnerable gap between RRP and ERP was wider from the RAA 90 (70,130) ms than BB 40 (20,80) ms, *P* = 0.01. The RACT was similar during the drive train from the RAA 123 (100,138) ms or the BB 119 (98,131) ms, *P* = 0.21 and increased significantly after the S2 10 ms above the ERP from the RAA 184 (174,228) ms, (*P* < 0.001 compared to RAA drive) or BB 165 (152,197) ms (*P* < 0.001 compared to BB drive). The RACT was significantly longer after the S2 10 ms above the ERP from the RAA 184 (174,228) ms compared to the BB 165 (152,197) ms (*P* = 0.03). The LACT was similar during the drive train from the RAA 32 (30,42) ms or the BB 31 (27,36) ms, *P* = 0.36 for difference and did not change after the S2 10 ms above the ERP from the RAA 36 (25,41) ms, (*P* = 0.76 compared to the drive from RAA) or the BB 33 (28,37) ms (*P* = 0.34 compared to the drive from BB). Complete RA and LA maps during S1 and S2 confirmed that RACT but not LACT prolonged during extrastimulation (*Figure 1*). Atrial tachycardia, AF or PACs were induced in 14/19 (74%) subjects during extrastimulation from the RAA and in 6/19 (32%) of subjects during extrastimulation from the BB, *P* = 0.02 for difference. All induced arrhythmias (84/84, 100%) occurred with an S2 in the vulnerable gap between the RRP and ERP, and the first beat of every induced arrhythmia (84/84,100%) occurred at the location of pacing (*Figure 1*).

## Discussion

Up to 65% of device-detected AF episodes are induced by CAP in the vulnerable gap.^7^ Despite new device algorithms and programming features designed to minimize CAP in the vulnerable gap, the frequency of CAP-induced AF remains high. A BB lead position may reduce the duration of the vulnerable gap compared to the RAA, thus reducing the probability that CAP will induce arrhythmia. Our study confirms in humans with a history of AF that the vulnerable gap is narrower during extrastimulation from the BB than the RAA, arrhythmia induction is less frequent during extrastimulation from the BB compared to RAA, and pacing-induced AF initiates with local re-entry near the site of extrastimulation.

## Limitations

The LACT was likely underestimated by catheter positioning; the subset of patients with complete LA and RA maps confirmed that conduction slowing during extrastimulation occurred exclusively in the RA. The findings are based on acute measurements and may not be transferable to chronic pacing.

## Data Availability

The data that support the findings of this study are available on request from the corresponding author. The data are not publicly available due to privacy restrictions.
